# The Impact of Implementation of a Clinically Integrated Problem-Based Neonatal Electronic Health Record on Documentation Metrics, Provider Satisfaction, and Hospital Reimbursement: A Quality Improvement Project

**DOI:** 10.2196/medinform.9776

**Published:** 2018-06-20

**Authors:** William Liu, Thomas Walsh

**Affiliations:** ^1^ Neonatology Golisano Children's Hospital of Southwest Florida Lee Health Fort Myers, FL United States; ^2^ Information Systems Lee Health Fort Myers, FL United States

**Keywords:** electronic health record, neonatal intensive care unit, NICU, physician documentation, Epic, SOI, ROM, CMI, APR-DRG, informatics

## Abstract

**Background:**

A goal of effective electronic health record provider documentation platforms is to provide an efficient, concise, and comprehensive notation system that will effectively reflect the clinical course, including the diagnoses, treatments, and interventions.

**Objective:**

The aim is to fully redesign and standardize the provider documentation process, seeking improvement in documentation based on ongoing All Patient Refined Diagnosis Related Group–based coding records, while maintaining noninferiority comparing provider satisfaction to our existing documentation process. We estimated the fiscal impact of improved documentation based on changes in expected hospital payments.

**Methods:**

Employing a multidisciplinary collaborative approach, we created an integrated clinical platform that captures data entry from the obstetrical suite, delivery room, neonatal intensive care unit (NICU) nursing and respiratory therapy staff. It provided the sole source for hospital provider documentation in the form of a history and physical exam, daily progress notes, and discharge summary. Health maintenance information, follow-up appointments, and running contemporaneous updated hospital course information have selected shared entry and common viewing by the NICU team. The interventions were to (1) improve provider awareness of appropriate documentation through a provider education handout and follow-up group discussion and (2) fully redesign and standardize the provider documentation process building from the native Epic-based software. The measures were (1) hospital coding department review of all NICU admissions and 3M All Patient Refined Diagnosis Related Group–based calculations of severity of illness, risk of mortality, and case mix index scores; (2) balancing measure: provider time utilization case study and survey; and (3) average expected hospital payment based on acuity-based clinical logic algorithm and payer mix.

**Results:**

We compared preintervention (October 2015-October 2016) to postintervention (November 2016-May 2017) time periods and saw: (1) significant improvement in All Patient Refined Diagnosis Related Group–derived severity of illness, risk of mortality, and case mix index (monthly average severity of illness scores increased by 11.1%, *P*=.008; monthly average risk of mortality scores increased by 13.5%, *P*=.007; and monthly average case mix index scores increased by 7.7%, *P*=.009); (2) time study showed increased time to complete history and physical and progress notes and decreased time to complete discharge summary (history and physical exam: time allocation increased by 47%, *P*=.05; progress note: time allocation increased by 91%, *P*<.001; discharge summary: time allocation decreased by 41%, *P*=.03); (3) survey of all providers: overall there was positive provider perception of the new documentation process based on a survey of the provider group; (4) significantly increased hospital average expected payments: comparing the preintervention and postintervention study periods, there was a US $14,020 per month per patient increase in average expected payment for hospital charges (*P*<.001). There was no difference in payer mix during this time period.

**Conclusions:**

A problem-based NICU documentation electronic health record more effectively improves documentation without dissatisfaction by the participating providers and improves hospital estimations of All Patient Refined Diagnosis Related Group–based revenue.

## Introduction

Wide-scale adoption of electronic health records (EHRs) became a national policy mandate in 2009, with allocation of significant health care dollars dependent on meaningful use implementation [1]. This has been justified by projected improvements in patient safety and health care quality [2].

The evidence for the benefits of EHR-based physician documentation is evolving. One challenge to implementation remains physician resistance, related to a myriad of operational and human factor barriers to creating the traditional physician medical note, including a perceived decrease in efficiency and an increased time expenditure [3,4].

The importance of physician documentation and the concept of problem-based documentation was originally championed by Lawrence Weed who recognized the importance of a systematic and comprehensive approach toward documenting the care of the complex intensive care patient with multisystem disease [5]. The translation of this complex process was accomplished primarily by handwritten or transcribed notes until the advent of the EHR. The EHR should efficiently collect, store, and display patient information in a way that will facilitate medical decision making and allow the provider to integrate this information, as reflected in his documentation [6].

All Patient Refined Diagnosis Related Groups (APR-DRGs) are the standard measure of provider medical documentation. Similar to the Centers for Medicare and Medicaid Medicare Severity Diagnosis Related Group inpatient prospective payment system, the APR-DRG provides a quantitative tool to measure accuracy and quality of physician documentation [7].

Our primary project goal was to fully redesign and standardize the provider documentation process, seeking improvement in documentation based on ongoing APR-DRG-based coding records, while maintaining noninferiority comparing provider satisfaction to our existing documentation process. We report the fiscal impact of improved documentation based on changes in expected APR-DRG-based hospital payments.

## Methods

### Background

In 2014-2015, all physician documentation at Lee Health in Fort Myers, FL, was transitioning from a dictation-based system to a full EHR platform using Epic software (Epic Systems Corporation, Verona, WI, USA). The Epic platform was already integrated into obstetrical and neonatal nursing, respiratory therapy, case management, pharmacy, laboratory, and imaging data entry, and the physician or provider component was a mandatory next step. Dictation-based documentation was the documentation method of choice. Between September 2015 and September 2016, the neonatal group transitioned to a combination of analog dictation with hospital-contracted transcriptionist documentation and a nonstandardized, individualized “out-of-box” Epic-based electronic documentation.

We began to redesign the provider documentation system in October 2015. We actively utilized Epic documentation capabilities as a “learning lab” for continuous improvement and refinement to achieve a final documentation system within Epic. From October 2015 to October of 2016, the providers utilized a shared electronic entry template, and some dictation continued as well. The shared template continued to use a clinical systems-based format for progress notes, and the entry was not problem-based. An ongoing feedback structure allowed providers to review benefits and drawbacks to note entry templates, smart phrases, general structure, and work flow. Real-time refinements based on this feedback allowed for a continuously evolving and improving documentation process.

In November 2016, the newly designed neonatal documentation system went live, and has continued through the study period of May 2017. Golisano Children’s Hospital level 2 and 3 neonatal services were provided primarily at HealthPark Medical Center neonatal intensive care unit (NICU), with a smaller level 2 care facility at Cape Coral Hospital Special Care Nursery. We chose not to include any further data in our analysis because the hospital NICU moved to a new facility in mid-May 2017.

### Aims and Goals

The aims and improvement goals of this project were to:

1. Improve provider awareness of appropriate documentation through a provider education handout and follow-up group discussion. We provided this education in August to September 2015, and an additional education with revision to the *International Classification of Diseases, Tenth Revision* (*ICD-10*) in January to February 2016.
2. Fully redesign and standardize the provider documentation process building from the native Epic-based software.
3. Create a comprehensive neonatal provider documentation system including the history and physical (H&P), progress note, and discharge summary that utilizes sharing and collaborative maternal and neonatal data entry by clinicians or staff in the obstetrical and neonatal work environments.
4. Improve provider care documentation as reflected by hospital 3M severity of illness (SOI), risk of mortality (ROM), and case mix index (CMI) scores. We did not have any target goals to increase diagnosis documentation, but rather sought to improve accuracy of documentation.
5. Achieve these goals without a negative perception of the new documentation process by the provider. This would be measured by time-based study by one provider and group survey after completion of the study.

### Methodology

We utilized a problem-based entry capability built into the hospital-wide vendor software to create a physician data entry structure that sought to efficiently manage the large data streams that occur in the NICU, enhance consistent and comprehensive identification of *ICD-10*-based diagnoses, streamline the use of an ongoing clinical summary form that can benefit all care providers with reduced redundancy of data entry, generation of a facile discharge summary, and provide a problem-based daily progress note that would encourage entry similar to the traditional physician problem-based SOAP (subjective, objective, assessment, and plan) note.

This project is a collaborative effort of the medical director of the NICU (WFL), all the neonatal providers, and a key hospital information system programmer (TW) and the Coding Documentation Improvement staff. The preintervention period was October 2015 to October 2016. Problem-based entry was a key component of the platform being created, and could not be implemented until the full configuration was in place. The postintervention period was November 2016 to May 2017.

Our initial intervention was provider education on recommended documentation optimization. In August to September 2015, the provider staff were given an orientation in optimal documentation (NICU Physician Documentation Guidelines) and encouraged to provide optimal and accurate documentation of patient clinical diagnoses. In October to November 2015, the hospital converted to *ICD-10* and revised versions of these guidelines were provided to the providers in January to February 2016.

**Figure 1:**
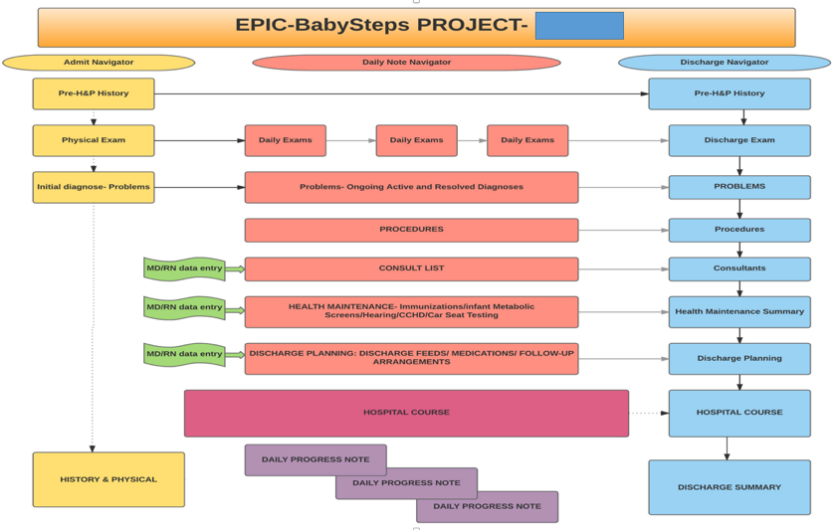
Development overview of components of history and physical (H&P), progress note, and discharge summary navigators.

Our second intervention was to design the neonatal EHR during the preintervention period: October 2015 to October 2016. “Go live” was November 14, 2016. Figure 1 illustrates the flow process for construction of provider documentation.

The following changes to the neonatal documentation process occurred. Over a 1-year period, through an ongoing clinician and information technology (IT) department collaboration, a more efficient and integrated neonatal EHR was constructed. Objectives for creating the tools focused on creating efficient and low effort data entry, and attempted to eliminate redundancies in documentation. Our perspective was that the quality of note readability facilitates communication of patient medical status.

Ongoing PDCA (plan-do-check-act) cycles refined the documentation structure incrementally, with monthly physician and IT design sessions. The neonatal provider team simultaneously constructed a facsimile of the H&P exam, progress note, and discharge summary templates, using these for real-time documentation, as well as reviewing and improving on variations on documentation strategies. Epic Sandbox simulations were conducted as the project matured to allow for more realistic assessments of effectiveness, as well as identification of limitations and needed revisions.

The following are some examples of the PDCA process. We improved efficiencies in data collection and sharing by initial identification of discrete data fields and implementation (eg, perinatal factors). This included early identification of missing data fields or incomplete data entry in the obstetrical admission process. This was followed by discussion with obstetricians and nursing staff, revision of data entry structure in Epic, and new workflow to capture the necessary data. This allowed for more efficient and consistent data entry for all maternal, antenatal, intrapartum, delivery room factors while the mother was being evaluated, and subsequent facile and efficient electronic transfer of data into the neonatal medical record. This would subsequently allow for transfer to the new neonatal H&P.

Another example of refinement was that redundant data collection was common to work flow with nursing and provider collection of health maintenance information (infant metabolic screening tests, critical congenital heart disease, pulse oximetry screening results, hearing test, car seat test, immunizations, physician follow-up appointments). As these needs became recognized in ongoing re-evaluations or study of implementation, IT added common entry fields that would be accessible and editable selectively by nursing and medical provider.

Our approach was to view constructing the discharge summary as beginning from birth. The discharge summary template was preconceived and a facsimile was utilized in the ongoing Epic documentation. We introduced the concept of a freestanding hospital course note that was created on admission. The provider entry into this hospital course note was intended to be incremental, with completion at the time of patient discharge. Other elements of clinical care relevant to discharge were preconfigured and completed during the hospitalization. Items such as hearing screen results, infant metabolic screens, and other routine testing were tracked and would be completed as the infant approached their discharge date.

We utilized problem-oriented charting. The transitional workflow encouraged providers to shift from system-oriented documentation to a habit of listing all relevant *ICD-10* diagnoses. We achieved efficient and consistent coding of diagnoses by identifying from the universe of *ICD-10* diagnoses, the most common neonatal diagnoses, which were included in a subset menu of common diagnoses.

The progress note template was created to allow for problem-based and system-bundled format for the progress note, maintaining the “SOAP” template familiar to providers.

The interim documentation did not utilize the problem-based Epic functionality until the actual program was rolled out in November 2016. This moved compliance from a primarily human vigilance paradigm to a structure and process that facilitated easier documentation compliance. Prior to our “go live” date, IT coordinated several in-service sessions using the hospital computer laboratory for all providers on the final documentation system.

### Measures and Analysis

The hospital clinical documentation improvement (CDI) team utilized 3M APR-DRG software, and a hospital-employed staff member reviewed each NICU admission, including all provider-generated neonatal patient documents (H&P, progress note, discharge summary, and consults) in the EHR from admission to discharge. This review was also inclusive of nursing notes, all flow sheets, laboratory values, and mother’s EHR. The 3M 360 software has an embedded natural language processor that helps the reviewer to identify potential diagnoses and sequence in order of severity. These diagnoses were manually verified by CDI staff. This encoding process was updated and resequenced until discharge. Utilizing the APR-DRG codes, the CDIS clinical logic algorithm generated a prioritized list of codes and calculated the SOI, ROM, and CMI. These results were reviewed by a CDI team staff member.

When comparing the preintervention and postintervention study periods, we used independent metrics to evaluate for any confounding variables in clinical acuity that might impact on the coding scores. These included monthly average length of stay, NICU total patient days, monthly admissions by birthweight category, and average daily census.

Average expected payment derived from the hospital finance software utilizing the calculated APR-DRG, SOI, ROM, and CMI provided the basis for hospital payment calculations. The hospital has a customized payer mix filter with predetermined payment estimations, that factor in third-party and government payer mix with contracted payments, and calculates an average expected payment for each patient. These algorithms remained consistent during the entire study period.

We employed a time study in which one provider (WFL) tracked his total time to complete clinical documentation, parsed into the time period before initiation of the new documentation system and after: defined as preintervention and postintervention. The preintervention period reflected time duration for completion of clinical documentation utilizing the traditional voice-dictated note, transcribed by a hospital-contracted transcriptionist. The postintervention period was composed of a random collection of EHR documentations using the new documentation process.

Provider perception was measured using a survey methodology. There were 13 providers (six neonatologists and seven neonatal nurse practitioners) who provided continuous documentation during the preintervention and through the postintervention periods; one neonatal nurse practitioner began employment during the study and was excluded from the survey. In June to July 2017, a SurveyMonkey-based survey was conducted for the 12 eligible providers. The survey was comprised of questions addressing subjective assessments of the H&P, progress note, and discharge summary processes, as well as an overall assessment of the change.

### Statistics

The clinical data for this study were obtained from the hospital Epic EHR utilizing an interface with Trendstar (The Shams Group) as well as clinical information compiled by an NICU-specific data analyst.

Trended data, using Minitab, was presented using statistical process control charting to visually present the impact of described interventions. Statistical process control uses entered data to describe common causes of variability, generating control limits, and identifying special causes of variation or statistically significant variance. Basic descriptive statistics were used, with *t* test for test of variance for continuous data using Microsoft Excel software or Minitab, with statistically significant variance defined as *P*<.05.

## Results

**Figure 2:**
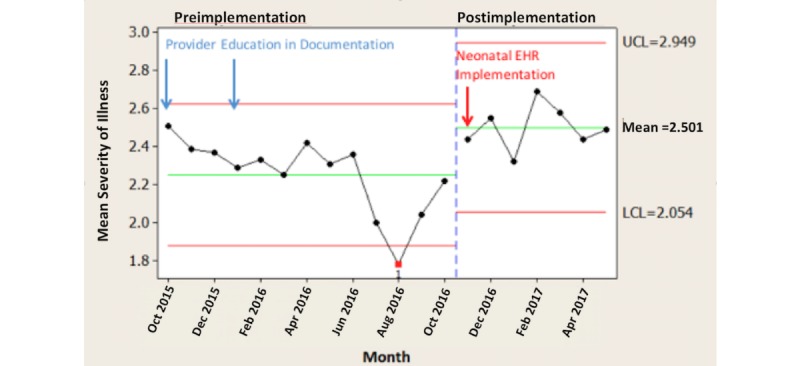
Severity of illness by pre-post neonatal electronic health record (EHR) implementation.

**Figure 3:**
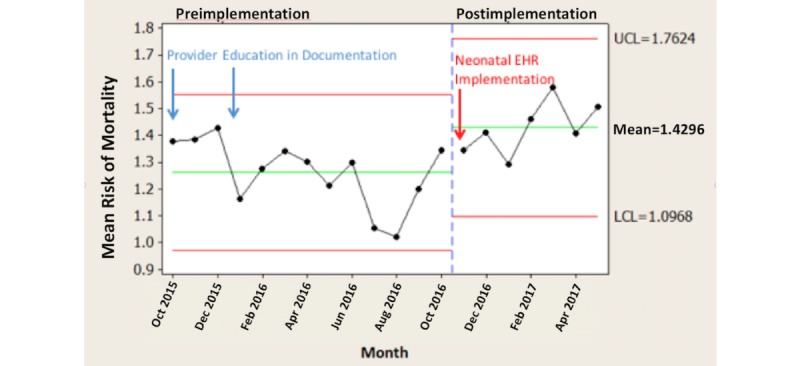
Risk of mortality by pre-post neonatal electronic health record (EHR) implementation.

**Figure 4:**
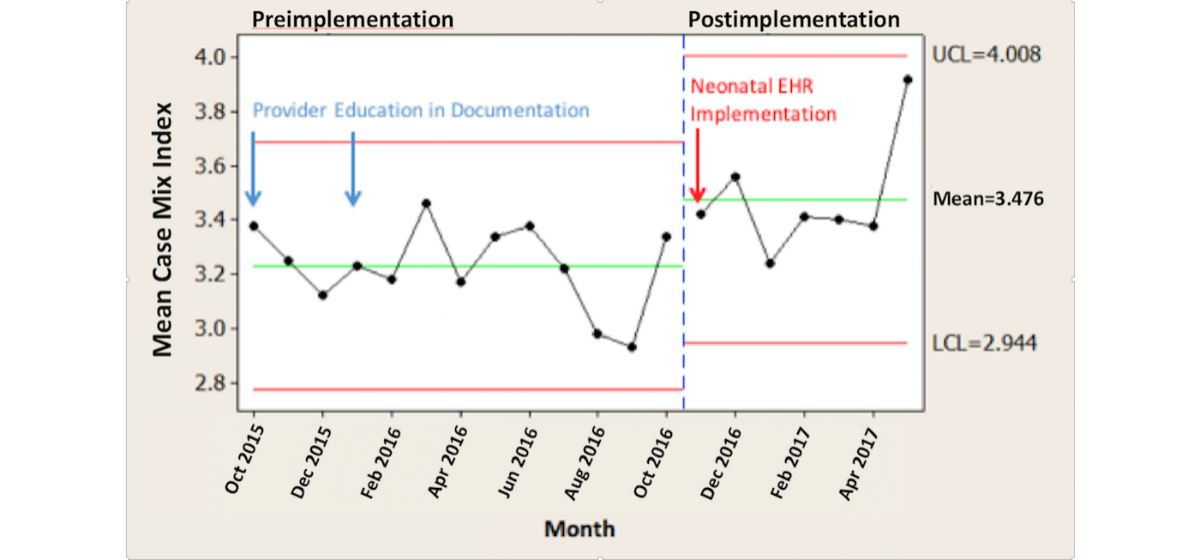
Case mix index by pre-post neonatal electronic health record (EHR) implementation.

Comparing the time periods October 2015 to October 2016 and November 2016 to May 2017, the monthly average SOI scores increased by 11.1% (*P*=.008; Figure 2), monthly average ROM scores increased by 13.5% (*P*=.007; Figure 3), and monthly average CMI scores increased by 7.7% (*P*=.009; Figure 4).

**Table 1:** Comparison of preintervention and postintervention groups in the neonatal intensive care unit (NICU) with potential confounders.

All values by month			NICU preintervention (Oct 2015-Oct 2016), mean (SD)	NICU postintervention (Nov 2016-May 2017), mean (SD)	*P* value
Severity of illness (SOI)			2.3 (0.2)	2.5 (0.1)	.008
Risk of mortality (ROM)			1.3 (0.1)	1.4 (0.1)	.007
Case mix index (CMI)			3.2 (0.2)	3.5 (0.2)	.009
**Acuity indicators**					
	Average length of stay		19.2 (1.3)	21.1 (3.9)	.12
	**Total patient days**		1329.9 (159.1)	1328.1 (116.6)	.98
		≤1000 g	351.0 (58.7)	282.3 (61.2)	.02
		1001-1500 g	189.6 (67.8)	237.0 (46.9)	.12
		1501-2500 g	442.4 (111.8)	431.7 (84.8)	.83
		>2500 g	346.9 (55.8)	377.1 (96.3)	.38
	**Admissions-total**		73.4 (7.8)	68.4 (9.5)	.22
		<1000 g	2.9 (2.4)	3.0 (1.2)	.94
		1000-1499 g	3.4 (1.5)	4.40 (3.3)	.34
		1500-1999 g	9.1 (2.9)	10.0 (2.9)	.51
		2000-2499 g	14.9 (3.0)	11.0 (3.9)	.03
		≥2500 g	43.2 (5.8)	40.0 (5.4)	.22
	**ADC^a^ (Level 2 and Level 3 combined)**		44.1 (4.1)	43.9 (3.5)	.88
		Level 2	25.0 (2.2)	25.3 (3.0)	.79
		Level 3	19.2 (4.3)	18.6 (2.7)	.75
	CCH-SCN^b^		5.7 (1.7)	4.7 (1.3)	.19

^a^ADC: average daily census.

^b^CCH-SCN: Cape Coral Hospital Special Care Nursery.

There was no evidence of change in clinical acuity during the comparison time periods (Table 1). There was a slight increase in the mean length of stay in the postintervention group, but not statistically significant (*P*=.12). There was no difference in three of four weight categories for total patient days. There were significantly more total patient days for babies weighing 1000 grams or less in the preintervention group (preintervention: mean 351.0, SD 58.7; postintervention: mean 282.3, SD 61.2; *P*=.02). There was no difference in four of five weight categories for total admissions per month. There were significantly more admissions for babies weighing between 2000 and 2499 grams in the preintervention group (preintervention: mean 14.9, SD 3.0; postintervention: mean 11.0, SD 3.9; *P*=.03). There was no difference in combined, level 3, or level 2 mean daily census.

**Figure 5:**
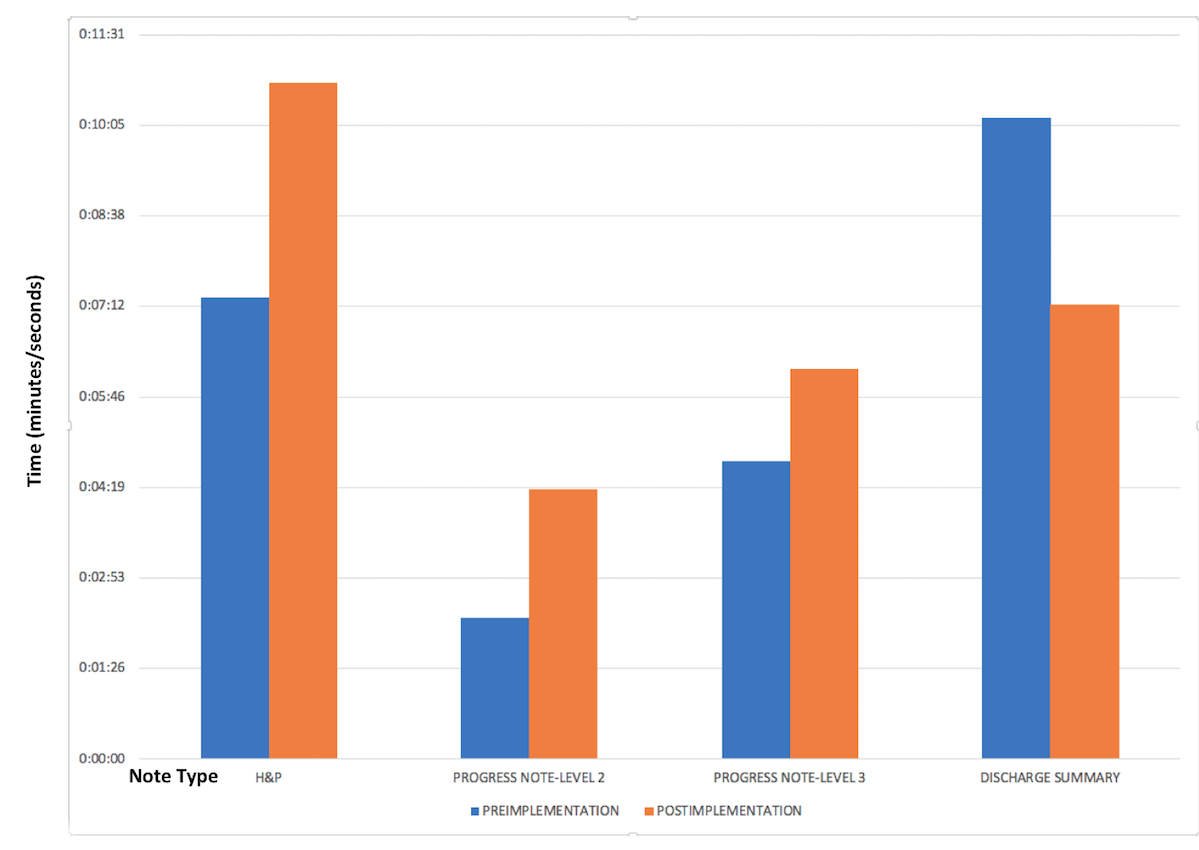
Time to complete history and physical (H&P) notes, levels 2 and 3 progress notes, and discharge summaries preintervention (dictation) and postintervention (electronic health record).

We assessed the impact on providers through a time expenditure study and a provider survey. One provider tracked his own time expenditure during the course of the project (Figure 5; Table 2). The H&P time allocation increased by 47% (*P*=.05). The progress note time allocation significantly increased by 91% (*P*<.001). The discharge summary time allocation significantly decreased by 41% (*P*=.03).

SurveyMonkey was used to poll all neonatal providers. Of 12 eligible providers, there was a 100% response to the survey, which was obtained in July 2017. Questions asked if the new process, as compared to the old process was... (based on a Likert scale) 1=much worse; 2=somewhat worse; 3=about the same; 4=better; 5=much better. Tables 3 and 4 summarize the results and are reported as percentage answering “somewhat worse” and percentage answering “better” or “much better.” No respondents answered “much worse” for any of the questions.

**Figure 6:**
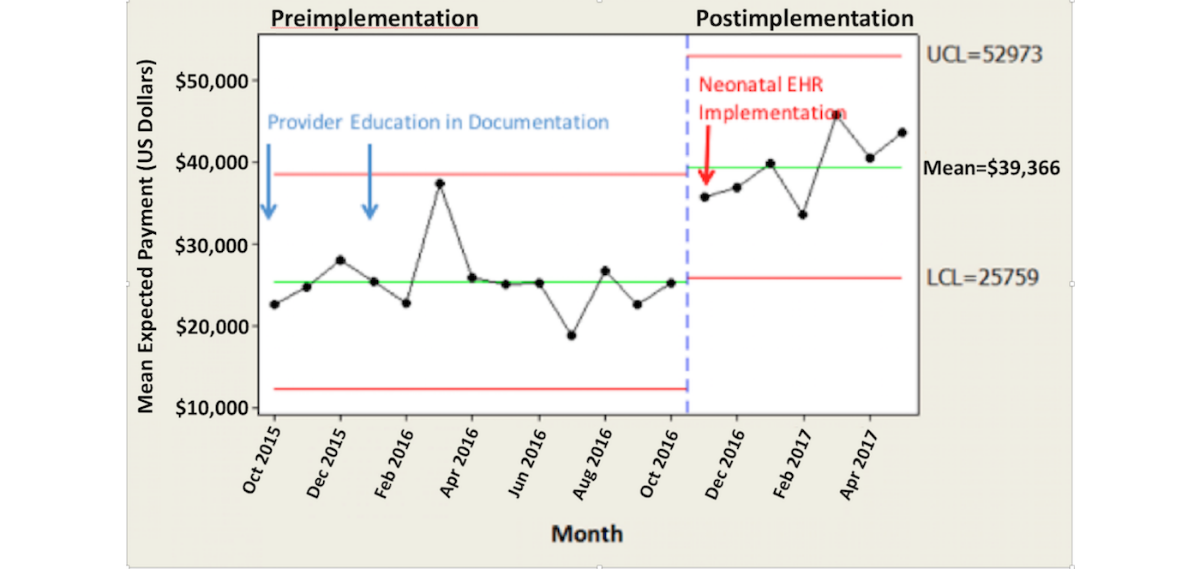
Trended average expected payment.

Hospital reimbursement was significantly improved. Comparing the preintervention and postintervention study periods, there was a US $14,020 per month per patient increase in average expected payment for hospital charges (*P*<.001; Figure 6). There was no difference in payer mix during this time period (Table 5).

**Table 2:** Documentation time: preintervention (primarily dictation method) compared to postintervention (electronic health record).

Note type	Preintervention		Postintervention		*P* value
	n	Time (min:sec)	n	Time (min:sec)	
History and physical (H&P)	12	7:20	10	10:45	.05
Progress note	31	2:15	24	4:18	<.001
Discharge summary	15	10:12	31	7:14	.03

**Table 3:** Survey of providers: summary (n=12).^a^

Survey question	History and physical (H&P), n (%)		Progress note, n (%)		Discharge summary, n (%)		Overall, n (%)	
	Somewhat worse	Better or much better	Somewhat worse	Better or much better	Somewhat worse	Better or much better	Somewhat worse	Better or much better
Ability to be comprehensive in my documentation	0 (0)	11 (92)	0 (0)	10 (83)	1 (8)	10 (83)	1 (3)	31 (86)
Ability to customize my documentation	0 (0)	11 (92)	0 (0)	11 (92)	0 (0)	10 (83)	0 (0)	32 (8)
Time allotment to accomplish this documentation	3 (25)	8 (67)	3 (25)	5 (42)	2 (17)	8 (67)	8 (22)	21 (58)

^a^Worse represents “somewhat worse” (no respondents answered “much worse” for any of the questions); better represents “better” and “much better.”

**Table 4:** Overall impression of providers (n=12).^a^

Overall impression	Somewhat worse, n (%)	Better or much better, n (%)
The overall documentation experience is...	0 (0)	11 (92)
My overall efficiency with documentation is...	2 (17)	7 (58)
My documentation accuracy (note reflects the true event) and validity (note states what I intended) is...	0 (0)	10 (83)
Documentation has made staff efficiency to collect information from multiple sources...	1 (8)	10 (83)
Documentation system has made the safety of patient care in the NICU^b^...	0 (0)	11 (92)

^a^Worse represents “somewhat worse” (no respondents answered “much worse” for any of the questions); better represents “better” and “much better.”

^b^NICU: neonatal intensive care unit.

**Table 5:** Payer mix comparison: preintervention and postintervention periods.

Differential payment estimations		Preintervention	Postintervention	*P* value
Average expected payment (US $), mean (SD)		25,346 (4291)	39,366 (4327)	<.001
**Payer mix, %**				
	Medicaid	71.6	72.2	.94
	Third party	26.4	26.3	.99
	Self-pay	0.8	0.4	.79

## Discussion

A problem-based NICU documentation EHR effectively improves documentation while avoiding dissatisfaction by the participating providers, and improves hospital estimations of APR-DRG-based revenue.

### Addressing Challenges to Neonatal Intensive Care Unit Documentation

The provider in the neonatal intensive care setting is challenged with a need to rapidly consolidate and integrate data that is being accumulated from several sources. The challenges to this in the NICU setting have been well described [8,9].

The NICU hospital course reflects a continuum of care with clinically relevant data collection beginning with a body of historical information that resides in the maternal (obstetrical) history: the woman’s preexisting medical conditions, her ongoing antepartum care, and the acute management of the delivering mother during the intrapartum care, which overlaps her admission and subsequent delivery. With the delivery of the newborn, the EHR should be populated with accessible information reflecting the newborn’s delivery room management and subsequent care in the NICU. This clinical course is composed of *ICD-10* and text-based diagnoses, assessments, and management interventions from not only the neonatal provider and specialist consultants, but also data entry by the NICU nurses, respiratory therapists, pharmacists, occupational therapists, dieticians, social workers, and case managers working with the patient’s family as well as a plethora of laboratory testing and imaging study results. The provider routinely assesses, consolidates, and integrates this information into a diagnosis and care plan.

We maintained an awareness of the original goals in a physician note. In 1968, Dr Weed [5] identified the importance of the problem-based note and of organizing clinical data. He outlined the characteristics of effective physician documentation and the need for annotation of active and resolved problem lists. The need to adhere to these principles is especially relevant when dealing with the intensive care patient with multisystem disease.

In October 2015, transcription services were still being used, as well as early participation in electronic entry of notes. The dictation system was a familiar tool that allowed the provider to generate a progress note rapidly, but the information was not digitally accessible. Our early electronic note entry remained essentially a word document without any structured identification of discrete data items. In both cases, the note structure, content, and identification of diagnoses were recorded inconsistently, with a high variation in structure among providers, and did not allow for data tracking or a linkage to a clinical data warehouse. There also was an inefficient collection and sharing of perinatal risk factors and clinical information with other NICU staff members due to the silo-based nature of documentation, with redundant workflows to acquire necessary information. A patient’s hospital course information was not easily accessible, and organization and completion of a discharge summary consequently was an inefficient and time-consuming task.

During the study period, we addressed these concerns with the following deliverables: (1) problem-oriented charting for documentation—Epic’s suite of documentation tools can be customized to meet complex patient workflows while also facilitating discrete data capture; (2) standard documentation templates for major neonatal diagnoses; (3) standard documentation templates for H&P, daily progress note, and discharge; (4) a suite of neonatal documentation smart links that encourage problem-oriented charting and optimize physician workflow efficiency; (4) the problem list at admission front loaded with gestational age-based problem recommendations giving the physician a quick and easy way to add multiple problem selections; (5) a problem preference list inclusive of the most common neonatal diagnoses; (6) a neonatal handoff to facilitate to-do list management and ease of provider handoffs of patient information; and (7) a structured course of care to manage pertinent historical data and to facilitate the expedited production of the discharge summary.

### Documentation Metrics

Illness classification systems function as a predictive model for federal resource allocation and to help track clinical outcomes. The APR-DRGs were developed in 1990 by 3M Health Information Systems jointly with the National Association of Children’s Hospitals and Related Institutions as the most comprehensive pediatric logic of any severity illness classification system, and were most recently updated to *ICD-10-CM*. By design, the APR-DRG system reflects completeness, accuracy, and specificity of documentation [7]. The EHR has been used to improve physician progress note documentation with documented improvement in *ICD-9* codes [10], and a provider-targeted educational intervention has been demonstrated to improve documentation as reflected in DRG-based coding metrics [11]. Although we were unable to see any improvement in our patient SOI, ROM, or CMI scores with our educational intervention, converting to a problem-based software platform within our hospital EHR resulted in significant improvement in documentation, without any demonstrable change in clinical acuity between the study periods. The increase in total patient days and a larger number of admissions of newborns weighing less than 1000 grams in the preintervention period would only skew toward less difference between the groups, suggesting a conservative appraisal of our improved documentation. Our findings suggest that strategies that are dependent on human diligence are much less effective than EHR-based process efficiencies.

### Provider Time Expenditure and Satisfaction

Physicians generally perceive that the EHR improves documentation, although many concerns are expressed [12]. Before initiation of this project, there was preexisting provider bias based on prior EHR experiences. These included a concern that there would be excessive time used for data entry and note production, and decreased productivity. An important goal was to decrease the time burden in generating a discharge summary (the NICU discharge summary often involves a 1-3 month length of stay requiring an extensive investment in time to review the hospital course, collect appropriate information, and then transcribe the summary). Our PDCA approach utilized continuous feedback from the end- user, allowing us to refine our templates and note design to better address these concerns.

In estimation of time expenditure, we recognized that many confounders existed in a time study involving more than one provider. After implementation of the EHR, each provider had their own unique approach toward document preparation and note entry. This degree of provider-specific variation did not lend itself well to comparisons among different providers. A single provider case study allowed for consistent measurement, adequate sampling, and provided qualitative insight into changes in time expenditure before and after implementation. These results cannot be taken as anything more than the impact on one individual provider. It did appear that in this one sample, the time required for producing the H&P and progress notes was increased, whereas the time for generating a comprehensive discharge summary was significantly decreased. We obtained overall insight by surveying the entire provider group.

Survey methodology is limited by design; however, in the context of our project, allowed for a systematic way to understand the provider experience. Our results are highly valid, reflecting the entire provider population that experienced the documentation process. In the context of a survey design, we were looking only for noninferiority or a perceptual equivalence, which is a more cautious approach to interpretation of our results. Complementing our case study of time allotment, the survey did not demonstrate a provider perception of increased time allotment for documentation. In fact, no participants chose the “much worse” option on the Likert scales, and there was generally a favorable response on all questions, including the overall documentation process, documentation efficiency, accuracy and validity, ability for staff to obtain and report clinical data, and overall perception of improved NICU safety. Our provider group adopted and adapted well to the new documentation process, without any evidence that the new process was worse, suggesting a better process than the one it replaced. We believe that early and ongoing provider engagement in the development process played a large role in ensuring greater provider satisfaction.

### Reimbursement

Projected EHR costs have remained an ongoing barrier to implementation, despite the emerging body of evidence that implementation of EHRs may lead to improved health outcomes with decreased medical errors and improved disease management [13,14].

There has been some evidence that there is a positive return on investment in adopting EHR in an ambulatory setting [15]. A challenge to this is that although adoption of EHRs may digitalize and standardize many of the clinical processes, they also impact on revenue-cycle functions. Poor awareness of this may have a negative impact on an organizations cash flow. Accurate and comprehensive capture of the clinical encounter is an important feature of an effective EHR, and reflects an effective merging of clinical and revenue-cycle operations with information technology [16].

Our documentation process focused not only on accurate and facile documentation of appropriate *ICD-10* diagnosis codes linked to concurrent supporting clinical elements, but on development, design, and entry interfaces based on a tight collaboration between the information systems programmer and the clinicians.

Clinical quality has become a major driver since the 2001 Institute of Medicine’s landmark publication “Crossing the Quality Chasm: A New Health System for the 21st Century” [17]. Concurrently, health care financing continues to move from traditional fee-for-service models to a more pay-for-performance, outcome-based reimbursement. Along these lines, hospital-based EHRs must provide the ability to effectively and efficiently document and track defined clinical interventions and outcomes on an individual and aggregated basis.

There is no doubt that the emergence of the EHR is transforming the way health care is delivered. The implementation of this change is taking place in the face of perceived provider dissatisfaction, decreased productivity, and uncertainty at the corporate level of the return on investment. The next step in health care reform involves improving value (as a function of outcomes divided by cost), especially as we are driven to improve clinical outcomes in the face of increasing fiscal accountability [18].

Of interest, is that this project was primarily focused on physician efficiency and documentation quality. The financials were never a primary goal. Our findings suggest that with a focus on high-quality care delivery, appropriate reimbursement gains will follow.

### Conclusions

Our project has demonstrated the clinical and fiscal effectiveness of a collaborative effort to create a more effective documentation system. There is clear noninferiority to our prior documentation process with respect to overall efficiency and a suggestion of an improved overall experience, as well as improved patient safety based on provider perception. We demonstrated that improved clinical documentation may also lead to improved hospital revenues, and clearly extends the dialog on the role of providers in addressing value-based care.

## Abbreviations

APR-DRG: All Patient Refined Diagnosis Related Group
CDI: clinical documentation improvement
CMI: case mix index
EHR: electronic health record
H&P: history and physical
ICD-10: International Classification of Diseases, Tenth Revision
IT: information technology
NICU: neonatal intensive care unit
PDCA: plan-do-check-act
ROM: risk of mortality
SOAP: subjective, objective, assessment, and plan
SOI: severity of illness
